# Distinct sleep-disordered breathing phenotypes in elderly patients with depressive disorder: links to hypoxemia severity and inflammatory burden

**DOI:** 10.3389/fpsyt.2026.1777040

**Published:** 2026-05-04

**Authors:** Jin-Xuan Zheng, Hui Jin, Shu-Jing Hu, Shan-Shan Zhu, Shun-Yao Xu

**Affiliations:** Department of Psychiatry, Wenzhou Seventh Peoples Hospital, Wenzhou, Zhejiang, China

**Keywords:** cluster analysis, depressive disorder, hypoxemia, obstructive sleep apnea-hypopnea syndrome, systemic inflammation

## Abstract

**Objective:**

To identify sleep-disordered breathing phenotypes in older adults with depressive disorder and obstructive sleep apnea-hypopnea syndrome (OSAHS) and to evaluate their associations with systemic inflammation.

**Methods:**

Elderly patients with depressive disorder and OSAHS were consecutively enrolled from January to December 2025. A Gower distance matrix was constructed and phenotypes were derived using partitioning around medoids (PAM; k-medoids), with k selected based on silhouette, elbow criteria, and clinical interpretability. Blood samples were collected the morning after PSG to measure serum high-sensitivity C-reactive protein (hs-CRP), interleukin-6 (IL-6), interleukin-1β (IL-1β), and tumor necrosis factor-α (TNF-α).

**Results:**

Among 198 participants, k = 2 was selected based on internal validity metrics (silhouette and elbow) and clinical interpretability. Compared with the lower-hypoxia/less-severe OSAHS phenotype (Cluster 1, n = 92), the high-hypoxia/severe OSAHS phenotype (Cluster 2, n = 106) had higher BMI, HAMD-17, and ESS, and more severe AHI/ODI/TS90 with a lower LSaO_2_. The high-hypoxia/severe OSAHS phenotype also showed higher hs-CRP, IL-6, IL-1β, TNF-α, WBC, neutrophils, and NLR. The inflammatory burden score was higher in the high-hypoxia/severe OSAHS phenotype (β = 1.10 SD unadjusted; β = 1.67 SD adjusted for age, sex, BMI, comorbidity, smoking, drinking, education, and MoCA; β = 1.45 SD further adjusted for HAMD-17 and ESS; all *P <* 0.001). In men (n = 135), PAM clustering similarly identified two phenotypes differentiated mainly by AHI/ODI, with selective elevations in IL-1β and neutrophil counts.

**Conclusions:**

The high-hypoxia/severe OSAHS phenotype in older adults with depressive disorder is independently associated with a higher systemic inflammatory burden.

## Introduction

Obstructive sleep apnea/hypopnea syndrome (OSAHS) is characterized by recurrent episodes of partial or complete upper-airway obstruction during sleep, leading to intermittent reductions in airflow and gas exchange (1). These events contribute to oxygen deprivation and impaired carbon dioxide elimination, and are commonly accompanied by snoring, sleep fragmentation, and daytime symptoms such as excessive sleepiness and impaired concentration (2). The global prevalence of OSAHS has been estimated to range from 9% to 38% (3), varying by region, sex, age distribution, diagnostic criteria, and the severity thresholds applied (4). Polysomnography (PSG) remains the diagnostic gold standard, providing objective quantification of respiratory events and oxygenation, and OSAHS is typically defined when the apnea–hypopnea index (AHI) exceeds five events per hour (5). However, OSAHS is a heterogeneous disorder with substantial variability in clinical presentation, polysomnographic profiles, and underlying pathophysiology; therefore, AHI alone does not fully capture the complexity and systemic consequences of the disease (6).

Depressive symptoms are common in OSAHS and may influence both symptom burden and clinical outcomes (7, 8). Sleep disruption, through sleep loss, altered sleep architecture, and fragmented sleep, may represent a plausible pathway linking OSAHS to depressive status, while improvements in sleep quality may mitigate depressive symptoms in affected individuals (9, 10). Importantly, late-life OSAHS often co-occurs with multimorbidity and age-related physiological vulnerability, potentially amplifying the clinical relevance of OSAHS–depression comorbidity in older adults (11).

Over the last decade, advances in understanding the pathophysiology, clinical presentation, systemic consequences, and treatment responses of OSAHS have made individualized OSAHS management increasingly plausible (12). In this context, phenotyping approaches that move beyond a “one-size-fits-all” strategy based solely on AHI have been proposed to improve risk stratification and support precision management (13, 14). Cluster analysis is a data-driven method that can identify relatively homogeneous subgroups within heterogeneous clinical data and has been increasingly applied in OSAHS populations to define clinically meaningful phenotypes with distinct symptom patterns and physiological profiles (6, 14, 15). Because the link between OSAHS and depressive symptoms is already well recognized, the key unresolved question in this comorbid geriatric population is not whether an association exists, but whether clinically meaningful sleep-disordered breathing phenotypes can be identified beyond conventional AHI-based grading and whether such phenotypes differ in biological burden. Because clinical phenotyping commonly integrates mixed data types (continuous PSG indices, categorical lifestyle factors, and ordinal symptom scales), distance-based unsupervised methods are well suited for this setting. Partitioning around medoids (PAM; k-medoids) clusters individuals around representative observed cases (medoids) and can be applied directly to a dissimilarity matrix, offering improved robustness to outliers compared with k-means (16, 17). We therefore used Gower’s distance, which accommodates mixed-type variables on a common 0–1 scale, and selected the number of clusters using internal validity indices (silhouette and elbow criteria) complemented by clinical interpretability (18). Nevertheless, phenotyping evidence remains limited in older adults with comorbid depressive disorder, a population in whom symptom interpretation may be particularly complex.

OSAHS is increasingly recognized as a systemic inflammatory condition, and inflammation may represent a key biological pathway through which sleep-disordered breathing contributes to downstream cardiometabolic and neuropsychiatric consequences (19). Chronic intermittent hypoxia related to OSAHS, together with sleep deprivation and fragmentation, can promote oxidative stress and inflammatory activation, leading to elevated circulating inflammatory mediators (2, 20). Pro-inflammatory cytokines such as high-sensitivity C-reactive protein (hs-CRP), interleukin-6 (IL-6), tumor necrosis factor-α (TNF-α), and interleukin-1β (IL-1β) have been reported to be increased in OSAHS, supporting the concept that inflammatory burden may vary across clinical and polysomnographic patterns of disease (21, 22). However, whether data-driven sleep-disordered breathing phenotypes among older adults with depressive disorder are associated with differential systemic inflammatory profiles remains insufficiently characterized.

Therefore, the present study aimed to identify sleep-disordered breathing phenotypes in elderly patients with depressive disorder and PSG-confirmed OSAHS using an unsupervised clustering approach, and to examine the associations between phenotype membership and systemic inflammatory burden. We further performed sensitivity analyses to assess clustering stability and to evaluate phenotype patterns within men, given the marked sex imbalance commonly observed in OSAHS cohorts.

## Materials and methods

### Ethics statement

The study protocol was approved by the Ethics Committee of our hospital. Written informed consent was obtained from all participants prior to enrollment.

### Participants

This study consecutively enrolled elderly OSAHS patients with depressive disorder from January to December 2025 in our hospital. All participants completed psychiatric assessment, PSG, and protocolized morning laboratory testing. Eligible participants met the following criteria (1): age ≥60 years (2); diagnosis of depressive disorder according to *Diagnostic and Statistical Manual of Mental Disorders, Fifth Edition* (DSM-5) (23) confirmed by trained psychiatrists (3); OSAHS confirmed by PSG with an apnea–hypopnea index (AHI) ≥5 events/h (24); and (4) completion of core assessments including depressive severity, sleepiness, cognitive screening, PSG indices, and blood sampling. Participants were excluded if they had any of the following (1): imminent suicide risk requiring emergency intervention or inpatient crisis management (2); psychotic depression or bipolar-spectrum disorder (current or past mania/hypomania) (3); severe neurological disease or other conditions that could compromise valid PSG acquisition or psychiatric evaluation (4); inability to complete key assessments or poor protocol adherence resulting in missing/invalid core data; or (5) acute infection or other acute inflammatory conditions at the time of blood collection, defined as any of the following: body temperature ≥ 38.0 °C within the preceding 48 hours; clinician-diagnosed acute infection requiring antimicrobial therapy within the preceding 14 days; systemic antibiotic use within the preceding 14 days; or markedly elevated inflammatory indices (hs-CRP >10 mg/L or WBC >10×10^9^/L). To reduce potential confounding of inflammatory biomarkers, we additionally excluded participants with documented systemic autoimmune/rheumatic disease or other chronic inflammatory disorders, as well as those receiving systemic corticosteroids, regular non-steroidal anti-inflammatory drugs, biologic immunomodulators, or other anti-inflammatory/immunosuppressive therapy at the time of enrollment. Current antidepressant and other psychotropic medication use was extracted from the medical record at enrollment and considered during clinical characterization; patients with newly initiated or majorly modified psychotropic regimens immediately preceding PSG/blood sampling were not eligible.

### Data collection

Depressive symptom severity was assessed using the 17-item Hamilton Depression Rating Scale (HAMD-17) (8). Daytime sleepiness was evaluated with the Epworth Sleepiness Scale (ESS) (25). Cognitive function was screened using the Montreal Cognitive Assessment (MoCA) (26). Demographic and lifestyle variables were obtained via structured interview and medical records, including age, sex, education level, current smoking, and current drinking. Current antidepressant and other psychotropic medications at the time of PSG were also recorded from the medication chart. Comorbidity burden was quantified as the number of physician-diagnosed chronic conditions (comorbidity count) documented in the medical record. Participants underwent attended overnight polysomnography (PSG) in the sleep laboratory using an Alice 6 system (Philips, Pittsburgh, PA, USA). PSG recordings with ≥4 h of interpretable data were considered acceptable for analysis. Raw signals were initially scored by the system software and then reviewed and manually corrected by trained sleep-respiratory physicians in accordance with the AASM Scoring Manual, Version 3, which was the contemporary scoring standard during the study period (27). Key PSG indices included the apnea–hypopnea index (AHI, events/h), oxygen desaturation index (ODI, events/h), percentage of total sleep time with SpO_2_ <90% (TS90, %), and lowest oxygen saturation (LSaO_2_, %).

### Clustering procedure and phenotype labeling

To identify sleep-disordered breathing phenotypes in elderly patients with depressive disorder and OSAHS, we followed the modified clustering framework proposed by Zhu et al. (28). The phenotyping feature set comprised 14 clinically interpretable variables spanning demographics, lifestyle, cognition, mood/sleepiness, comorbidity, and OSAHS severity/hypoxemia: age, sex, body mass index (BMI), education level, current smoking, current drinking, Montreal Cognitive Assessment (MoCA) score, Hamilton Depression Rating Scale-17 (HAMD-17) score, Epworth Sleepiness Scale (ESS) score, comorbidity count, apnea–hypopnea index (AHI), oxygen desaturation index (ODI), percentage of total sleep time with SpO_2_ < 90% (TS90), and lowest oxygen saturation (LSaO_2_). Because the feature set included a mixture of continuous and categorical variables, we computed pairwise dissimilarities using the Gower distance, which rescales each continuous variable to a 0–1 range and combines variable-wise dissimilarities across mixed data types into an overall distance (16). Clustering was then performed using partitioning around medoids (PAM; k-medoids), an unsupervised method that selects k representative observations (medoids) by minimizing total within-cluster dissimilarity; compared with k-means, PAM is less sensitive to extreme values and is well-suited to distance matrices derived from mixed-type data (17, 29). Candidate solutions (k = 2–8) were evaluated using the mean silhouette coefficient and an elbow curve based on total within-cluster dissimilarity (PAM cost) (18), and the final k was selected based on these metrics together with clinical interpretability. Cluster membership was visualized using a two-dimensional multidimensional scaling (MDS) projection of the Gower distance matrix. After cluster assignment, groups were characterized by between-cluster comparisons of all variables and by within-cluster correlation patterns, and phenotype labels were assigned *post hoc* according to the dominant distinguishing clinical and polysomnographic features, prioritizing hypoxemia burden (ODI, TS90, LSaO_2_), respiratory event frequency (AHI), daytime sleepiness (ESS), depressive symptom severity (HAMD-17), and comorbidity load. As a redundancy-reduction sensitivity analysis, clustering was repeated using AHI and TS90 as representative measures of respiratory event frequency and sustained hypoxemia burden, respectively, and agreement with the full 14-variable solution was quantified using the adjusted Rand index (ARI) and the proportion of participants retaining the same cluster membership (30).

### Blood sampling and specimen processing

Venous blood was collected the following morning immediately after overnight PSG, with participants remaining fasting. Phlebotomy was performed between 07:00 and 09:00 within a fixed 2-hour window for all participants. Serum was separated within 1 hour of collection, aliquoted, and stored at −80 °C until batch analysis. Primary systemic inflammatory markers were high-sensitivity C-reactive protein (hs-CRP), interleukin-6 (IL-6), interleukin-1β (IL-1β), and tumor necrosis factor-α (TNF-α). Blood cell indices, including white blood cell count (WBC), neutrophils, lymphocytes, and platelets, were measured using an automated hematology analyzer in the hospital laboratory. Two derived ratios were computed: Neutrophil-to-lymphocyte ratio (NLR) = neutrophils/lymphocytes, and Platelet-to-lymphocyte ratio (PLR) = platelets/lymphocytes.

### Statistical analysis

Continuous variables are presented as mean ± SD or median (IQR), and categorical variables as n (%). Between-cluster comparisons used Welch’ s t-test or the Mann-Whitney U test for continuous variables, and the chi-square or Fisher’ s exact test for categorical variables, as appropriate. Spearman rank correlations were calculated in the overall cohort and within each cluster. Multivariable associations with inflammatory/hematologic outcomes were examined using heteroskedasticity-robust linear regression (HC3). Cluster differences (Cluster 2 vs Cluster 1) were evaluated in sequential models adjusting for age, sex, BMI, comorbidity count, smoking, drinking, education, and MoCA, with additional adjustment for HAMD-17 and ESS. Respiratory event and hypoxemia metrics (AHI, ODI, TS90, and LSaO_2_; per 1 SD) were entered one at a time into fully adjusted models including cluster membership; an ODI × cluster interaction term and cluster-stratified models were used to assess effect modification and consistency. All tests were two-sided with *P <* 0.05 indicating statistical significance.

## Results

### Identification of two phenotypes in elderly OSAHS patients with depression

**Figure 1:**
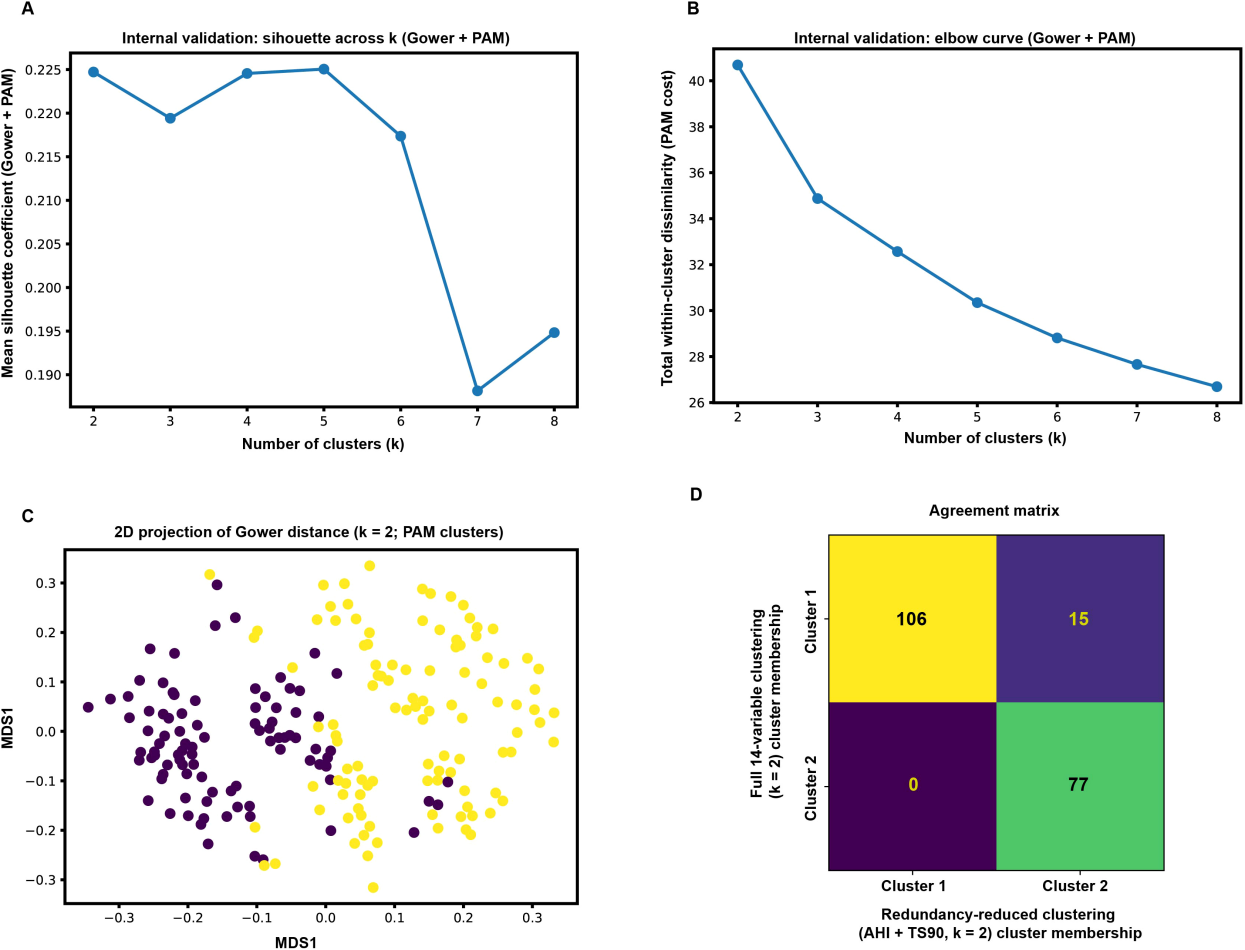
Clustering workflow, internal validation, and robustness of Gower distance + PAM phenotyping in elderly patients with depressive disorder and OSAHS. **(A)** Mean silhouette coefficient across candidate numbers of clusters (k = 2–8) for Gower distance–based PAM (k-medoids) phenotyping. **(B)** Elbow curve depicting total within-cluster dissimilarity (PAM cost) across k = 2–8. **(C)** Two-dimensional multidimensional scaling (MDS) projection of the Gower distance matrix for the selected k = 2 solution; each point represents one participant and is colored by phenotype membership (Cluster 2 corresponds to the high-hypoxia/severe OSAHS phenotype). **(D)** Agreement matrix comparing the redundancy-reduced AHI+TS90 clustering solution with the full 14-variable solution (k = 2; labels aligned to maximize agreement); overall agreement was 183/198 (92.4%), with an adjusted Rand index (ARI) of 0.72. PAM, partitioning around medoids; MDS, multidimensional scaling; ARI, adjusted Rand index.

Using Gower distance with partitioning around medoids (PAM; k-medoids), candidate solutions (k = 2–8) were internally validated. The mean silhouette score indicated modest but non-trivial separation, with the highest silhouette observed at k = 2 (≈0.225) and a comparable value around k = 5, whereas silhouette dropped notably at higher k (**Figure 1A**). The elbow curve based on total within-cluster dissimilarity (cost) showed a marked reduction from k = 2 to k = 3, followed by progressively smaller gains with additional clusters, supporting a parsimonious solution (**Figure 1B**). Considering internal validity metrics together with interpretability, k = 2 was selected. A two-dimensional MDS projection of the Gower distance matrix showed partial separation with expected overlap between the two clusters, consistent with clinical phenotypes that lie on continuous severity spectra (**Figure 1C**). As a redundancy-reduction sensitivity analysis, clustering was repeated using AHI and TS90 as representative measures of event frequency and hypoxic burden, respectively; cluster assignments showed high agreement with the full 14-variable solution (ARI = 0.72), with 92.4% (183/198) of participants retaining the same cluster membership. Reassignment occurred primarily from the lower-hypoxia cluster to the higher-hypoxia cluster (15/198), with no reverse migration (**Figure 1D**).

### Two distinct clinical phenotypes in elderly OSAHS patients with depression

As shown in **Table 1**, compared with the lower-hypoxia/less-severe OSAHS phenotype (Cluster 1), the high-hypoxia/severe OSAHS phenotype (Cluster 2) was younger (68.95 ± 6.34 vs. 74.58 ± 5.92 years; *P <* 0.001), had a higher BMI (28.32 ± 2.03 vs. 27.04 ± 2.45 kg/m²; *P <* 0.001), more severe depressive symptoms (HAMD-17: 28.73 ± 4.36 vs. 25.09 ± 3.93; *P <* 0.001), and greater daytime sleepiness (ESS: 10.99 ± 3.69 vs. 8.77 ± 3.51; *P <* 0.001). PSG indices indicated markedly more severe sleep-disordered breathing and hypoxemia in the high-hypoxia/severe OSAHS phenotype, including higher AHI and ODI, higher TS90, and lower nadir oxygen saturation (all *P <* 0.001). Sex distribution differed substantially, with the high-hypoxia/severe OSAHS phenotype being predominantly male (96.2% vs. 35.9%; *P <* 0.001). Education level did not differ significantly (*P =* 0.276), and MoCA score and comorbidity count were comparable between phenotypes (*P =* 0.905 and *P =* 0.810, respectively). Current antidepressant use and other psychotropic medication use were common in both phenotypes, with no significant between-cluster differences (all *P* > 0.05). Current smoking and drinking were more prevalent in the high-hypoxia/severe OSAHS phenotype (both *P <* 0.001).

**Table 1:** Two distinct clinical phenotypes in elderly OSAHS patients with depression.

Variable	Cluster 1 (N = 92)	Cluster 2 (N = 106)	*P*
**Age, years**	74.58 ± 5.92	68.95 ± 6.34	< 0.001
**Sex**			< 0.001
Female, n (%)	59 (64.1%)	4 (3.8%)	
Male, n (%)	33 (35.9%)	102 (96.2%)	
**BMI, kg/m²**	27.04 ± 2.45	28.32 ± 2.03	< 0.001
**Education level**			0.276
≤6 years, n (%)	58 (63.0%)	70 (66.0%)	
7–12 years, n (%)	31 (33.7%)	28 (26.4%)	
>12 years, n (%)	3 (3.3%)	8 (7.5%)	
**MoCA score**	21.17 ± 2.98	21.23 ± 3.20	0.905
**Current smoking**			< 0.001
No, n (%)	89 (96.7%)	49 (46.2%)	
Yes, n (%)	3 (3.3%)	57 (53.8%)	
**Current drinking**			< 0.001
No, n (%)	84 (91.3%)	60 (56.6%)	
Yes, n (%)	8 (8.7%)	46 (43.4%)	
**Number of comorbidities**	1.39 ± 0.88	1.42 ± 1.07	0.810
**HAMD-17 scores**	25.09 ± 3.93	28.73 ± 4.36	< 0.001
**Current antidepressant use, n (%)**	63 (68.5%)	79 (74.5%)	0.349
**Stable antidepressant regimen, n (%)**	58 (63.0%)	73 (68.9%)	0.381
**Any other psychotropic medication use, n (%)**	29 (31.5%)	39 (36.8%)	0.433
**ESS score**	8.77 ± 3.51	10.99 ± 3.69	< 0.001
**AHI, events/h**	27.35 [20.68, 36.17]	43.00 ± 11.22	< 0.001
**ODI, events/h**	26.28 ± 12.49	38.40 ± 8.95	< 0.001
**TS90, %**	7.20 [3.85, 11.72]	14.70 ± 7.40	< 0.001
**LSaO_2_, %**	80.64 ± 5.51	75.04 ± 6.31	< 0.001

The lower-hypoxia/less-severe phenotype corresponds to Cluster 1, and the high-hypoxia/severe phenotype corresponds to Cluster 2. Continuous variables are presented as mean ± SD if approximately normally distributed, or median [IQR] otherwise. Categorical variables are presented as n (%). P values were derived from Welch’s t-test for normally distributed continuous variables, Mann–Whitney U test for non-normal continuous variables, and χ² test (or Fisher’s exact test when appropriate) for categorical variables. Current antidepressant use refers to antidepressant treatment being taken at enrollment. Stable antidepressant regimen refers to no newly initiated antidepressant treatment and no major change in antidepressant class or dose immediately before PSG and blood sampling. Any other psychotropic medication use includes hypnotics/anxiolytics, antipsychotic augmentation, or mood stabilizers. BMI, body mass index; MoCA, Montreal Cognitive Assessment; HAMD-17, 17-item Hamilton Depression Rating Scale; ESS, Epworth Sleepiness Scale; AHI, apnea–hypopnea index; ODI, oxygen desaturation index; TS90, percentage of total sleep time with SpO_2_ < 90%; LSaO_2_, lowest oxygen saturation.

Bold text indicates main variables or section headings.

### Higher systemic inflammation in cluster 2

Cluster 2 showed a substantially higher systemic inflammatory burden than Cluster 1 (**Table 2**). hs-CRP, IL-6, IL-1β, and TNF-α were all significantly elevated in Cluster 2 (all *P <* 0.001). Hematologic inflammatory indices were also higher, including WBC and neutrophil counts (both *P <* 0.001). NLR was higher in Cluster 2 (*P <* 0.001), whereas lymphocyte count and platelet count did not differ significantly (*P =* 0.134 and *P =* 0.120). PLR showed a borderline difference (*P =* 0.053).

**Table 2:** Systemic inflammatory and hematologic profiles across sleep-disordered breathing phenotypes.

Variable	Cluster 1 (N = 92)	Cluster 2 (N = 106)	*P*
hs-CRP (mg/L)	2.75 ± 1.18	3.78 ± 1.08	< 0.001
IL-6 (pg/mL)	2.23 ± 0.87	3.19 ± 0.89	< 0.001
IL-1β (pg/mL)	1.19 ± 0.55	1.59 ± 0.47	< 0.001
TNF-α (pg/mL)	5.90 ± 1.83	7.67 ± 1.71	< 0.001
WBC	5.67 ± 1.38	7.08 ± 1.32	< 0.001
Neutrophils	3.53 ± 0.98	4.41 ± 0.72	< 0.001
Lymphocytes	1.73 ± 0.37	1.81 ± 0.34	0.134
Platelets	232.89 ± 43.60	221.74 ± 56.75	0.120
NLR	2.02 [1.59, 2.59]	2.54 ± 0.69	< 0.001
PLR	136.78 [117.64, 160.57]	127.58 ± 41.57	0.053

The lower-hypoxia/less-severe phenotype corresponds to Cluster 1, and the high-hypoxia/severe phenotype corresponds to Cluster 2. Data are presented as mean ± SD or median [IQR] as appropriate. Between-phenotype comparisons used Welch’s t-test or the Mann–Whitney U test, as appropriate. hs-CRP, high-sensitivity C-reactive protein; IL-6, interleukin-6; IL-1β, interleukin-1β; TNF-α, tumor necrosis factor-α; WBC, white blood cell count; NLR, neutrophil-to-lymphocyte ratio; PLR, platelet-to-lymphocyte ratio.

### Association between hypoxia phenotype and inflammatory burden in the full cohort

Using HC3-robust linear regression in the full cohort (n = 198), the high-hypoxia/severe OSAHS phenotype was associated with a higher inflammatory burden score compared with the low-hypoxia phenotype (**Table 3**). In the unadjusted model, inflammatory burden was higher by 1.10 SD (β = 1.10; 95% CI 0.92–1.28; *P <* 0.001). The association remained significant after adjustment for age, sex, BMI, comorbidity count, current smoking, current drinking, education level, and MoCA score (Model 2: β = 1.67 SD; 95% CI 1.43–1.92; *P <* 0.001). Further adjustment for depressive symptom severity and daytime sleepiness (HAMD-17 and ESS) attenuated the estimate but the association persisted (Model 3: β = 1.45 SD; 95% CI 1.17–1.73; *P <* 0.001).

**Table 3:** Stepwise HC3-robust linear regression of the inflammatory burden score by sleep-disordered breathing phenotype in the full cohort (n = 198).

Model	Adjustments	β (SD units), 95% CI	P value
Model 1	Unadjusted	1.10 (0.92, 1.28)	< 0.001
Model 2	Age, sex, BMI, comorbidity, smoking, drinking, education, MoCA	1.67 (1.43, 1.92)	< 0.001
Model 3	Model 2 + HAMD-17 + ESS	1.45 (1.17, 1.73)	< 0.001

The outcome was an inflammatory burden score defined as the mean z-score of log1p-transformed hs-CRP, IL-6, IL-1β, and TNF-α plus the z-scores of WBC, neutrophils, and NLR. β estimates are reported in SD units for the high-hypoxia/severe phenotype (Cluster 2) versus the lower-hypoxia/less-severe phenotype (Cluster 1), with 95% confidence intervals, using heteroskedasticity-robust standard errors (HC3). Model 1 was unadjusted. Model 2 adjusted for age, sex, BMI, comorbidity count, current smoking, current drinking, education level, and MoCA score. Model 3 additionally adjusted for HAMD-17 and ESS. BMI, body mass index; MoCA, Montreal Cognitive Assessment; HAMD-17, 17-item Hamilton Depression Rating Scale; ESS, Epworth Sleepiness Scale; hs-CRP, high-sensitivity C-reactive protein; IL, interleukin; TNF-α, tumor necrosis factor-α; WBC, white blood cell count; NLR, neutrophil-to-lymphocyte ratio.

### Male-only clustering identified a higher event-frequency/desaturation phenotype broadly aligned with the main analysis

**Figure 2:**
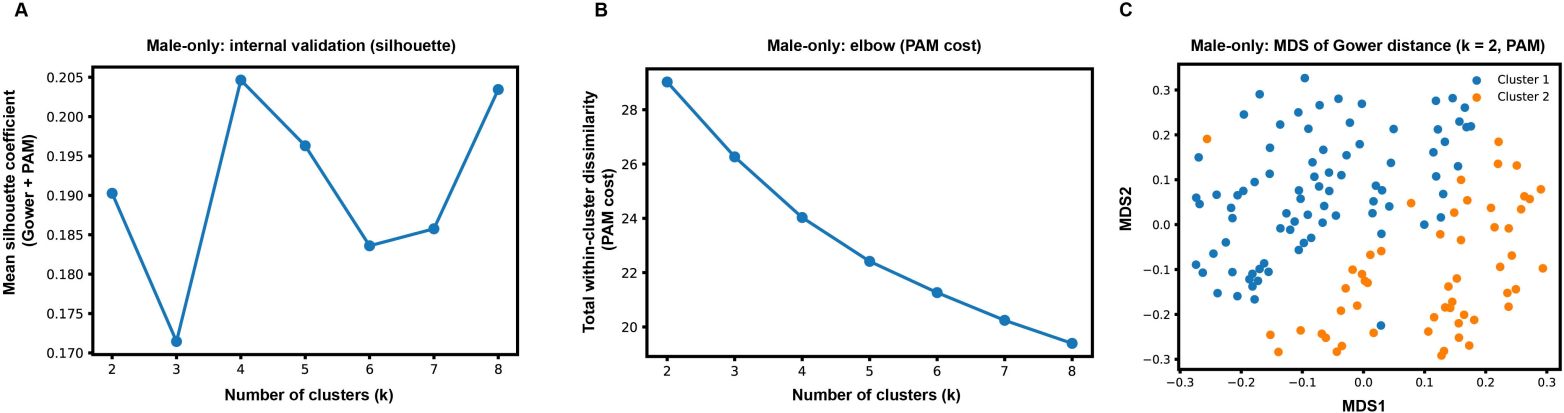
Internal validation and visualization of male-only Gower distance + PAM phenotyping. **(A)** Mean silhouette coefficient across candidate numbers of clusters (k = 2–8) for male-only phenotyping using Gower distance with PAM (k-medoids). **(B)** Elbow curve showing total within-cluster dissimilarity (PAM cost) across k = 2–8. **(C)** Two-dimensional MDS projection of the Gower distance matrix for the selected k = 2 solution; each point represents one male participant and is colored by phenotype membership (Cluster 1: high event-frequency/desaturation phenotype; Cluster 2: lower event-frequency/desaturation phenotype). PAM, partitioning around medoids; MDS, multidimensional scaling.

In a male-only sensitivity analysis (n = 135), Gower distance with partitioning around medoids (PAM; k-medoids; k = 2) identified two phenotypes with adequate sample sizes: a high event-frequency/desaturation phenotype (Cluster 1, n = 81) and a lower event-frequency/desaturation phenotype (Cluster 2, n = 54) (**Figure 2**; **Table 4**). Compared with the lower event-frequency/desaturation phenotype, the high event-frequency/desaturation phenotype was older (74.00 [69.00, 78.00] vs. 66.00 [62.25, 72.50] years; *P <* 0.001) and had a lower BMI (27.88 ± 1.91 vs. 28.71 ± 2.24 kg/m²; *P =* 0.026). The lower event-frequency/desaturation phenotype showed markedly higher prevalences of current smoking (81.5% vs. 14.8%) and drinking (68.5% vs. 18.5%) (both *P <* 0.001), and a higher comorbidity count (2.00 [1.00, 3.00] vs. 1.00 [0.00, 1.00]; *P <* 0.001). Depressive severity did not differ significantly between phenotypes (HAMD-17, *P =* 0.180), whereas daytime sleepiness was higher in the high event-frequency/desaturation phenotype (ESS: 12.00 [8.00, 14.00] vs. 9.00 ± 3.88; *P =* 0.003). Current antidepressant use and other psychotropic medication use were common in both male phenotypes, with no significant between-cluster differences (all *P* > 0.05). Regarding polysomnography, the high event-frequency/desaturation phenotype showed more severe event frequency (AHI: 42.32 ± 14.01 vs. 35.30 [30.97, 41.12] events/h; *P =* 0.008) and oxygen desaturation burden (ODI: 38.60 [32.10, 44.10] vs. 33.28 ± 9.38 events/h; *P =* 0.015), while TS90 and LSaO_2_ did not differ significantly (*P =* 0.073 and *P =* 0.104, respectively), suggesting that the male-only solution was primarily differentiated by event frequency and desaturation indices rather than by sustained hypoxemia exposure. In inflammatory profiling, IL-1β and neutrophil counts were higher in the high event-frequency/desaturation phenotype (*P =* 0.014 and *P =* 0.026), whereas hs-CRP, TNF-α, lymphocytes, platelets, and PLR were comparable; IL-6, WBC, NLR, and platelet count showed borderline differences (*P =* 0.054–0.084) (**Table 4**), supporting a consistent direction of greater OSAHS physiological stress being linked to higher inflammatory activity, although the dominant separating axis in men was event-frequency/desaturation rather than sustained hypoxemia.

**Table 4:** Male-only sleep-disordered breathing phenotypes identified by Gower distance + PAM clustering (k = 2) and their clinical, polysomnographic, and inflammatory profiles (n = 135).

Variable	Cluster 1 (N = 81)	Cluster 2 (N = 54)	P
**Age, years**	74.00 [69.00, 78.00]	66.00 [62.25, 72.50]	< 0.001
**BMI, kg/m²**	27.88 ± 1.91	28.71 ± 2.24	0.026
**Education level**			0.784
≤ 6 years, n (%)	53 (65.4%)	35 (64.8%)	
7–12 years, n (%)	23 (28.4%)	17 (31.5%)	
> 12 years, n (%)	5 (6.2%)	2 (3.7%)	
**MoCA score**	21.00 [19.00, 23.00]	21.33 ± 3.08	0.364
**Current smoking**			< 0.001
No, n (%)	69 (85.2%)	10 (18.5%)	
Yes, n (%)	12 (14.8%)	44 (81.5%)	
Current drinking			< 0.001
No, n (%)	66 (81.5%)	17 (31.5%)	
Yes, n (%)	15 (18.5%)	37 (68.5%)	
**Comorbidity count**	1.00 [0.00, 1.00]	2.00 [1.00, 3.00]	< 0.001
**HAMD-17 scores**	28.00 [24.00, 32.00]	27.00 [25.00, 30.75]	0.18
**Current antidepressant use, n (%)**	59 (72.8%)	37 (68.5%)	0.596
**Stable antidepressant regimen, n (%)**	54 (66.7%)	35 (64.8%)	0.821
**Any other psychotropic medication use, n (%)**	27 (33.3%)	16 (29.6%)	0.657
**ESS score**	12.00 [8.00, 14.00]	9.00 ± 3.88	0.003
**Polysomnographic indices**			
**AHI (events/h)**	42.32 ± 14.01	35.30 [30.97, 41.12]	0.008
**ODI (events/h)**	38.60 [32.10, 44.10]	33.28 ± 9.38	0.015
**TS90 (%)**	13.00 [10.10, 18.20]	9.70 [7.10, 17.73]	0.073
**LSaO_2_ (%)**	75.83 ± 7.14	77.57 ± 5.24	0.104
**hs-CRP (mg/L)**	3.51 ± 1.30	3.44 [2.79, 3.96]	0.276
**IL-6 (pg/mL)**	3.04 ± 1.10	2.74 ± 0.85	0.074
**IL-1β (pg/mL)**	1.58 ± 0.56	1.38 ± 0.40	0.014
**TNF-α (pg/mL)**	7.36 ± 2.15	6.89 ± 1.64	0.156
**WBC (×10^9^/L)**	7.06 [5.76, 7.59]	6.55 [5.97, 7.20]	0.054
**Neutrophils (×10^9^/L)**	4.37 [3.84, 4.97]	4.04 ± 0.61	0.026
**Lymphocytes (×10^9^/L)**	1.76 ± 0.34	1.82 ± 0.35	0.335
**Platelets (×10^9^/L)**	216.57 ± 52.75	232.81 ± 53.21	0.084
**NLR**	2.48 ± 0.72	2.30 ± 0.52	0.081
**PLR**	128.14 ± 40.30	133.10 ± 41.33	0.491

In the male-only sensitivity analysis, the high event-frequency/desaturation phenotype corresponds to Cluster 1, and the lower event-frequency/desaturation phenotype corresponds to Cluster 2. Continuous variables are presented as mean ± SD if approximately normally distributed, or median [IQR] otherwise. Categorical variables are presented as n (%). P values were derived from Welch’s t-test for normally distributed continuous variables, Mann–Whitney U test for non-normal continuous variables, and χ² test (or Fisher’s exact test when appropriate) for categorical variables. Current antidepressant use refers to antidepressant treatment being taken at enrollment. Stable antidepressant regimen refers to no newly initiated antidepressant treatment and no major change in antidepressant class or dose immediately before PSG and blood sampling. Any other psychotropic medication use includes hypnotics/anxiolytics, antipsychotic augmentation, or mood stabilizers. BMI, body mass index; MoCA, Montreal Cognitive Assessment; HAMD-17, 17-item Hamilton Depression Rating Scale; ESS, Epworth Sleepiness Scale; AHI, apnea–hypopnea index; ODI, oxygen desaturation index; TS90, percentage of total sleep time with SpO_2_ < 90%; LSaO_2_, nadir oxygen saturation; hs-CRP, high-sensitivity C-reactive protein; IL, interleukin; TNF-α, tumor necrosis factor-α; WBC, white blood cell count; NLR, neutrophil-to-lymphocyte ratio; PLR, platelet-to-lymphocyte ratio.

Bold text indicates main variables or section headings.

## Discussion

In this cohort of older adults with depressive disorder and PSG-confirmed OSAHS, an unsupervised, Gower-distance–based PAM approach identified two clinically interpretable sleep-disordered breathing phenotypes characterized primarily by differences in hypoxemia burden and sleep-disordered breathing severity. The high-hypoxia/severe OSAHS phenotype (Cluster 2) exhibited substantially higher systemic inflammatory activity, spanning hs-CRP, IL-6, IL-1β, and TNF-α, as well as hematologic indices (WBC, neutrophils, and NLR), than the lower-hypoxia phenotype. Importantly, the association between phenotype membership and an aggregate inflammatory burden score remained robust after sequential adjustment for demographic factors, adiposity, comorbidity burden, lifestyle factors, education/cognition, and further adjustment for depressive severity and daytime sleepiness, supporting that phenotype-related hypoxemia severity is independently associated with systemic inflammation. These results are consistent with meta-analytic and mechanistic evidence that OSAHS is accompanied by upregulated inflammatory signaling, including elevated hs-CRP, IL-6, and TNF-α (31).

A mechanistically coherent explanation for the observed phenotype–inflammation gradient is the central role of intermittent hypoxia and downstream immune activation. Recurrent oxygen desaturations promote oxidative stress, endothelial dysfunction, and activation of pro-inflammatory transcriptional programs (notably NF-κB) and innate immune pathways, which can upregulate cytokines such as IL-6 and TNF-α and thereby amplify systemic inflammatory tone (32, 33). In addition, emerging immunology syntheses emphasize that OSAHS’s hallmark stressors, including intermittent hypoxia and sleep fragmentation, drive immune dysregulation through pathways such as HIF-1α, NF-κB, and the NLRP3 inflammasome, providing a plausible route for the IL-1β signal observed here (34). Collectively, these pathways offer biological plausibility for why the phenotype with greater hypoxemic stress exhibited higher cytokine levels and a higher composite inflammatory burden.

The finding of higher WBC/neutrophils and NLR in the high-hypoxia phenotype is also biologically consistent. Intermittent hypoxia and sympathetic activation can shift leukocyte trafficking, promote myeloid predominance, and enhance neutrophil activation, with NLR serving as a convenient marker of subclinical systemic inflammation (35). Notably, prior meta-analyses support an association between OSAHS and elevated NLR, and interventional work suggests that effective OSAHS treatment can improve inflammatory markers, supporting partial reversibility of at least part of the inflammatory burden (36, 37). This aligns with our observation that the phenotype defined by more severe hypoxemia and desaturation burden showed higher NLR and neutrophil counts.

All participants had depressive disorder, and depression itself is associated with heightened inflammatory signaling, particularly in older adults, potentially creating a background of age-related low-grade inflammation on which OSAHS-related hypoxic stress adds incremental inflammatory load (38). Moreover, conceptual models linking sleep disturbance to inflammation and depressive symptomatology provide a plausible bidirectional framework: OSAHS-related sleep disruption/hypoxia may increase inflammation, and inflammation may, in turn, exacerbate depressive symptoms and fatigue (39). Within this framework, our results suggest that heterogeneity in sleep-breathing physiology, especially hypoxemia burden, may contribute to meaningful between-patient differences in inflammatory burden even within a clinically defined depressive-disorder cohort.

In the male-only analysis, PAM clustering again produced two phenotypes, but separation was driven more by event frequency/desaturation indices (AHI/ODI) than by sustained hypoxemia exposure (TS90/LSpO_2_). This difference is not necessarily discordant with the main analysis; rather, it likely reflects sex-restricted variance in oxygenation metrics, different confounding structures (e.g., markedly different smoking/drinking prevalence and comorbidity burden across male clusters), and reduced power for certain biomarkers. The selective elevations in IL-1β and neutrophils in the higher event-frequency/desaturation cluster remain biologically plausible in light of inflammasome/innate immune activation frameworks in OSAHS (34). Taken together, the male-only findings support broad alignment with the main phenotype signal while underscoring that the specific physiologic axis differentiating clusters may shift when the cohort is restricted by sex.

Several limitations should be considered when interpreting the results: (1) Although enrollment and testing were protocolized, the analysis is primarily cross-sectional; causal inference cannot be established and residual confounding remains possible (2). Generalizability may be limited by the single-center design (3). The silhouette value suggests modest separation, which is common in clinical phenotyping where traits lie on continua; nonetheless, it underscores that phenotypes overlap and should be interpreted as probabilistic groupings (4). The study did not include a non-OSAHS control group, a non-depressed OSAHS group, or a healthy comparison group; therefore, the relative contributions of OSAHS, depressive disorder, and aging-related background inflammation cannot be fully disentangled (5). The cohort showed marked sex imbalance, particularly in the high-hypoxia/severe phenotype, and although a male-only sensitivity analysis was feasible, a female-only clustering analysis was not performed because of the limited female sample size and the very small number of women in Cluster 2; accordingly, extrapolation of phenotype structure to women should be made with caution.

## Conclusions

In older adults with depressive disorder and OSAHS, an unsupervised phenotyping approach identified a high-hypoxia/severe OSAHS phenotype that was independently associated with substantially greater systemic inflammatory burden, spanning cytokines and hematologic inflammatory indices. These results support hypoxemia-informed phenotyping as a clinically meaningful framework for identifying higher-inflammatory-risk subgroups and motivate longitudinal and interventional studies to test whether targeted OSAHS treatment attenuates inflammation and improves downstream outcomes, particularly in the high-hypoxia phenotype.

## Data Availability

The raw data supporting the conclusions of this article will be made available by the authors, without undue reservation.
